# CDK4/6‐Inhibitor in Kombination mit Aromatasehemmer wirksam bei metastasiertem Hidradenokarzinom

**DOI:** 10.1111/ddg.15723_g

**Published:** 2025-08-11

**Authors:** Valerie Glatzel, Dirk Tomsitz, Michaela Maurer, Simone Schneider, Stefan Brunner, Nadia Harbeck, Lucie Heinzerling

**Affiliations:** ^1^ Klinik und Poliklinik für Dermatologie und Allergologie der LMU München; ^2^ Brustzentrum und Onkologische Tagesklinik der Frauenklinik der LMU München; ^3^ Medizinische Klinik und Poliklinik I – Kardiologie der LMU München

**Keywords:** Abemaciclib, CDK4/6‐Inhibitoren, Hidradenokarzinom, Letrozol, Myokarditis, Schweißdrüse, abemaciclib, CDK4/6‐inhibitors, Hidradenocarcinoma, letrozole, myocarditis, sweat gland

Sehr geehrte Herausgeber,

Das Hidradenokarzinom ist ein sehr seltener maligner Tumor der Schweißdrüse,^1^ der sich als erythematöser oder hautfarbener Knoten meist an der Kopfhaut oder im Nacken präsentiert. Es gibt nur wenige Fallberichte und bisher keine zugelassenen Therapien. CDK4/6‐Inhibitoren, wie Abemaciclib, Ribociclib und Palbociclib, sind wirksam bei Hormonrezeptor‐positivem, HER2‐negativem metastasierenden Mammakarzinom.

Dieser Fallbericht beschreibt die wirksame Therapie aus einem CDK4/6‐Inibitor und einem Aromatasehemmer bei einer 75‐jährigen Patientin mit metastasiertem Hidradenokarzinom (Abbildung 1).

**ABBILDUNG 1:**
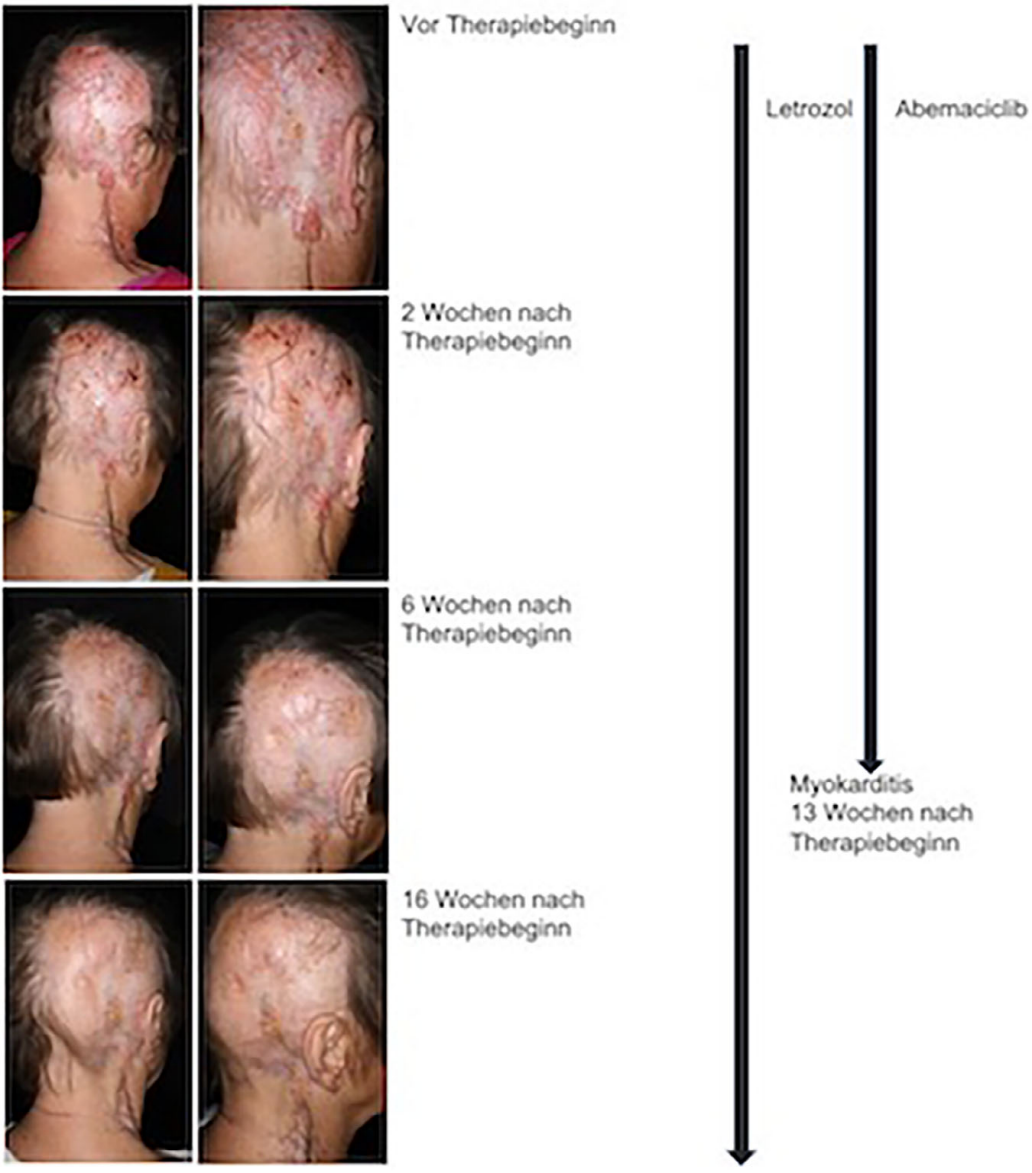
Klinischer Verlauf des Therapieansprechens des Hidradenokarzinoms auf die Behandlung mit einem CDK4/6‐inhibtor und dem Aromatasehemmer Letrozol.

Die Patientin stellte sich mit einem ausgedehnten Tumor des Kapillitiums sowie einer zervikalen Lymphknotenmetastase vor. Da Hidradenokarzinome eine spezifische Expression von Tumormarkern aufweisen, wurde eine immunhistochemische Untersuchung durchgeführt. Der Tumor war positiv für Östrogen‐ und Progesteronrezeptoren und negativ für HER2; CK7 und GATA3 waren positiv, während CK5, CK6 und PD‐L1 nicht exprimiert wurden (Abbildung 2).

**ABBILDUNG 2:**
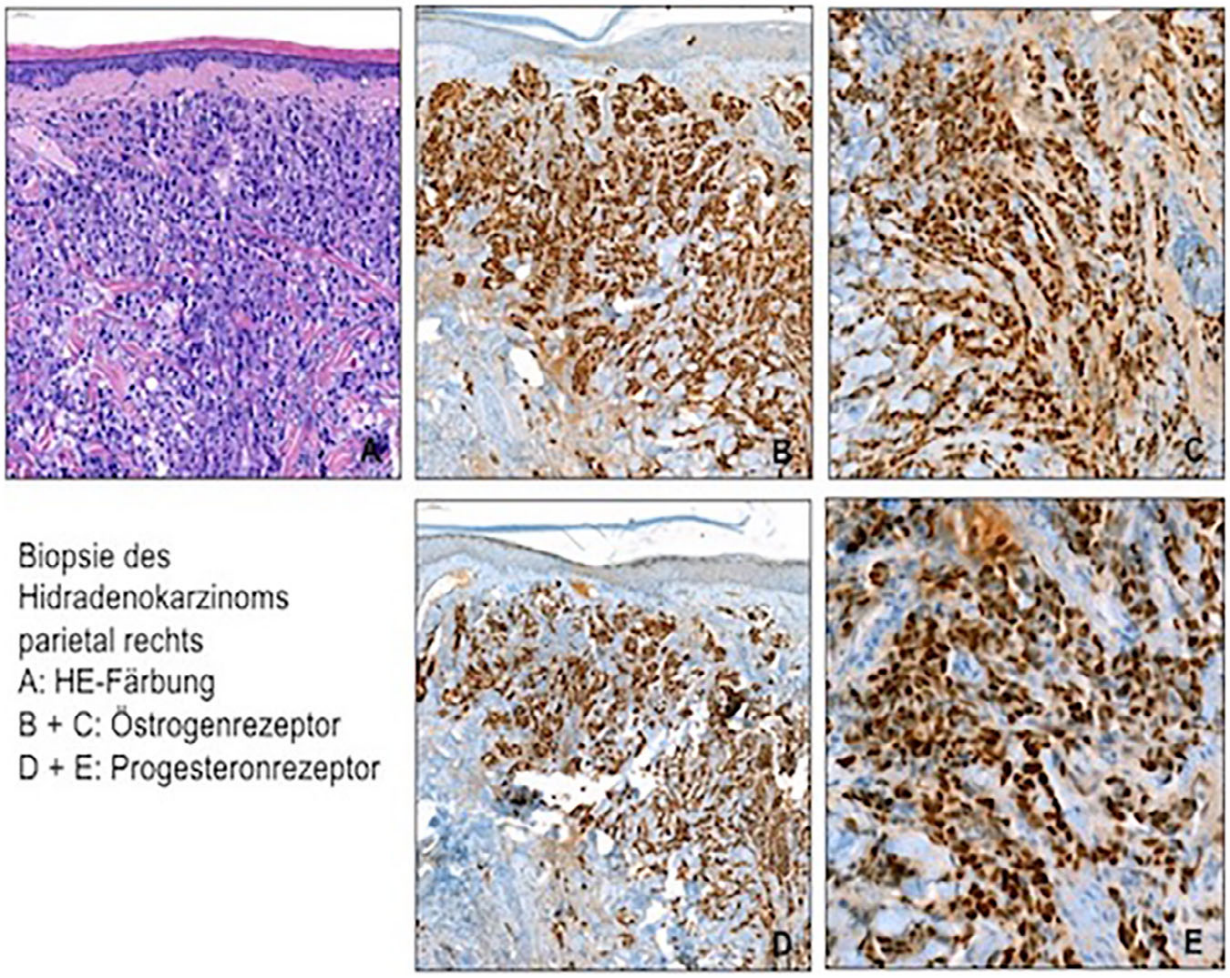
Histologie eines duktalen Adenokarzinoms des Kapillitiums, identifiziert als Hidradenokarzinom, inklusive positiver Östrogen‐ und Progesteronrezeptorfärbung. Der Tumor ist positiv für CK7 und GATA3, aber negativ für CK5, CK6 und PD‐L1.

Pathologisch konnte eine Brustkrebsmetastase nicht ausgeschlossen werden, aber die klinische Beurteilung und eine Mammographie wiesen kein Mammakarzinom nach. Eine CT‐Untersuchung bestätigte die zervikale Lymphknotenmetastase ohne Anzeichen für Fernmetastasen.

Insgesamt wurden fünf Exzisionen sowie die Resektion von retroaurikulären subkutanen Metastasen und zervikalen Lymphknotenmetastasen im Level V durchgeführt. Anschließend erfolgte eine Radiotherapie (50 Gy, 60 Gy und 64 Gy). Nach erneutem Auftreten des Tumors und zervikaler Metastasen wurden diese chirurgisch entfernt und eine *neck dissection* durchgeführt.

*Next‐generation sequencing* mittels *TruSight™ Oncology 500 molecular assay on the Illumina platform* ergaben keine zielführenden Mutationen, Genfusionen oder Amplifikationen. Die Tumormutationslast lag bei 2,4 Mutationen pro Mb und der Mikrosatellitenstatus des Tumors war stabil. Aufgrund der starken Expression der Hormonrezeptoren wurde analog zur Behandlung des hormonrezeptorpositiven Mammakarzinoms eine Therapie mit einem CDK4/6‐Inhibitor und dem Aromatasehemmer Letrozol begonnen. Nach Voruntersuchungen inklusive Elektro‐ und Echokardiographie leiteten wir Abemaciclib 150 mg 1‐0‐1 in Kombination mit Letrozol 2 mg 1‐0‐0 ein.

Bereits 2 Wochen nach Therapiebeginn zeigte sich ein signifikantes klinisches Ansprechen (Abbildung 1). Bei den regelmäßigen Laborkontrollen war die glomeruläre Filtrationsrate reduziert und der Kreatininwert erhöht. Die Zahl der Leukozyten und Erythrozyten war mit 3,19 G/l und 3,30 T/l erniedrigt. Eine bei Therapiebeginn auftretende Diarrhoe konnte mit Loperamid gut kontrolliert werden. Nach 13 Wochen klagte die Patientin über stechenden Brustschmerz. Die kardiologische Diagnostik in der Notaufnahme ergab eine durch Abemaciclib induzierte Myokarditis mit Troponinämie und moderater Reduktion der linksventrikulären Ejektionsfraktion. Deshalb wurde der CDK4/6‐Inhibitor beendet. Da eine Myokarditis für Palbociclib nicht beschrieben ist, wäre ein Klassenwechsel möglich gewesen. Aufgrund des guten Ansprechens führten wir aber die Therapie nur mit Letrozol fort. Die Patientin befindet sich weiterhin in klinischer Remission mit einer Nachbeobachtungszeit von 40 Wochen.

Schweißdrüsenkarzinome sind schwierig zu diagnostizieren, es gibt verschiedene Subtypen, teils überlappende Merkmale und keine etablierte systemische Therapie.^2^ Die Primärtherapie besteht aus einer lokalen Exzision, postoperativer Radiotherapie und/oder Chemotherapie.^1^, ^2^, ^3^

Dennoch rezidivieren und metastasieren die Tumoren häufig. Antiandrogene Therapie eines Androgenrezeptor‐positiven Schweißdrüsenkarzinoms,^3^ und eine antiöstrogene Therapie einen östrogenrezeptorpositiven Schweißdrüsenkarzinoms waren in einzelnen Fällen erfolgreich.^4^

Bei unserer Patientin wurde analog zur Behandlung von fortgeschrittenem Brustkrebs ein CDK4/6‐Inhibitor in Kombination mit der antiöstrogenen Therapie mit Letrozol begonnen. CDK4/6‐Inhibitoren greifen in das Zellwachstum ein, indem sie die Phosphorylierung des Retinoblastom‐Proteins verhindern und den Übergang von der G1‐Phase zur S‐Phase hemmen.

Leukozytopenie, Thrombozytopenie und Anämie können auftreten, Kreatinin muss kontrolliert werden. Thromboembolien wurden beschrieben.^5^, ^6^ Obwohl weder Bestandteil der Fachinformation noch beschrieben in der Literatur, ist die Myokarditis in der Sicherheitsdatenbank des Herstellers beschrieben. Daher ist dies die erste Publikation einer Abemaciclib‐induzierten Myokarditis.

Das Schweißdrüsenkarzinom ist ein sehr seltener Tumor ohne Standardtherapie, besonders bei metastasierter oder rezidivierender Erkrankung. Dieser Fallbericht zeigt die erfolgreiche Behandlung mit einem CDK4/6‐Inhibitor in Kombination mit einem Aromatasehemmer.

## DANKSAGUNG

Wir möchten Prof. Dr. med. Lars E. French (Klinik und Poliklinik für Dermatologie und Allergologie, LMU München, Deutschland), Dr. med. Alexander König (Brustzentrum, Klinik und Poliklinik für Frauenheilkunde und Geburtshilfe, LMU München) sowie Prof. Dr. med. Frederick Klausch (Institut für Pathologie, LMU München) für ihren Beitrag zu diesem Fall unseren aufrichtigen Dank aussprechen.

Open access Veröffentlichung ermöglicht und organisiert durch Projekt DEAL.

## INTERESSENKONFLIKT

Keiner.
